# Detection of Urinary Exosomal HSD11B2 mRNA Expression: A Useful Novel Tool for the Diagnostic Approach of Dysfunctional 11β-HSD2-Related Hypertension

**DOI:** 10.3389/fendo.2021.681974

**Published:** 2021-08-23

**Authors:** Domenica De Santis, Annalisa Castagna, Elisa Danese, Silvia Udali, Nicola Martinelli, Francesca Morandini, Mariangela Veneri, Lorenzo Bertolone, Oliviero Olivieri, Simonetta Friso, Francesca Pizzolo

**Affiliations:** ^1^Unit of Internal Medicine, Department of Medicine, University of Verona, Verona, Italy; ^2^Section of Clinical Biochemistry, University and Azienda Ospedaliera Universitaria Integrata of Verona, Verona, Italy

**Keywords:** apparent mineralocorticoid excess, 11β-hydroxysteroid dehydrogenase type 2, urinary cortisol metabolites ratio, urinary exosomal mRNA, Droplet Digital PCR, HSD11B2, essential hypertension

## Abstract

**Objective:**

Apparent mineralocorticoid excess (AME) is an autosomal recessive disorder caused by the 11β-hydroxysteroid dehydrogenase type 2 (11β-HSD2) enzyme deficiency, traditionally assessed by measuring either the urinary cortisol metabolites ratio (tetrahydrocortisol+allotetrahydrocortisol/tetrahydrocortisone, THF+5αTHF/THE) or the urinary cortisol/cortisone (F/E) ratio. Exosomal mRNA is an emerging diagnostic tool due to its stability in body fluids and its biological regulatory function. It is unknown whether urinary exosomal HSD11B2 mRNA is related to steroid ratio or the HSD11B2 662 C>G genotype (corresponding to a 221 A>G substitution) in patients with AME and essential hypertension (EH).

**Aim of the Study:**

To detect and quantify HSD11B2 mRNA from urinary exosomes in samples from family members affected by AME and EH, and to evaluate the relationship between exosomal HSD11B2 mRNA, steroid ratio, 662C>G genotype, and hypertension.

**Methods:**

In this observational case–control study, urinary steroid ratios and biochemical parameters were measured. Urinary exosomes were extracted from urine and exosomal HSD11B2 mRNA was quantified by Droplet Digital PCR (ddPCR). B2M (β-2 microglobulin) gene was selected as the reference housekeeping gene.

**Results:**

Among family members affected by AME, exosomal urinary HSD11B2 mRNA expression was strictly related to genotypes. The two homozygous mutant probands showed the highest HSD11B2 mRNA levels (median 169, range 118–220 copies/µl) that progressively decreased in 221 AG heterozygous with hypertension (108, range 92–124 copies/µl), 221 AG heterozygous normotensives (23.35, range 8–38.7 copies/µl), and wild-type 221 AA subjects (5.5, range 4.5–14 copies/µl). Heterozygous hypertensive subjects had more HSD11B2 mRNA than heterozygous normotensive subjects. The F/E urinary ratio correlated with HSD11B2 mRNA copy number (*p* < 0.05); HSD11B2 mRNA strongly decreased while THF+5αTHF/THE increased in the two probands after therapy. In the AME family, HSD11B2 copy number correlated with both F/E and THF+5αTHF/THE ratios, whereas in EH patients, a high F/E ratio reflected a reduced HSD11B2 mRNA expression.

**Conclusions:**

HSD11B2 mRNA is detectable and quantifiable in urinary exosomes; its expression varies according to the 662 C>G genotype with the highest levels in homozygous mutant subjects. The HSD11B2 mRNA overexpression in AME could be due to a compensatory mechanism of the enzyme impairment. Exosomal mRNA is a useful tool to investigate HSD11B2 dysregulation in hypertension.

## Introduction

Apparent mineralocorticoid excess (AME) is a rare autosomal recessive disorder caused by the 11β-hydroxysteroid dehydrogenase type 2 (11β-HSD2) enzyme deficiency that consequently leads to an activation of the mineralocorticoid receptor (MR) not mediated by aldosterone (1). The MR is characterized by similar affinity for both aldosterone and cortisol, but in spite of the higher plasma concentration of cortisol as compared to that of aldosterone (about 100-fold), specific MR activation by aldosterone is physiologically guaranteed because of the specific 11β-HSD2 enzymatic activity. The enzyme, in fact, converts the active steroids cortisol and corticosterone respectively into their inactive metabolites, i.e., cortisone and 11-dehydrocorticosterone, thus allowing a functional selectivity of aldosterone to activate MR and regulate the epithelial sodium transport (2, 3). An impaired 11β-HSD2 function leads to an accumulation of active steroid forms in the renal distal tubular cells, with subsequent MR activation and sodium retention with the development of a clinical syndrome characterized by sodium retention, hypokalemia, salt-dependent hypertension, low renin, and suppressed aldosterone concentrations (1, 4–7). The activity of the 11β-HSD2 enzyme can be estimated either by measuring the urinary cortisol metabolites ratio (tetrahydrocortisol+allotetrahydrocortisol/tetrahydrocortisone, THF+5αTHF/THE) or by measurement of serum or urinary free cortisol/cortisone ratio (F/E). An increase in urinary (THF+5αTHF)/THE ratio or urinary F/E indicates a decreased 11β-HSD2 activity.

AME is a rare disease *per se* although it was hypothesized that a mild reduction of 11β-HSD2 activity can have a role also in low renin essential hypertension (EH). Milder forms of AME, recently named as “non-classic” AME, are at higher prevalence than classic AME, have a different phenotype and genotype, and are mainly related only to a partial enzymatic deficiency (8–10).

Urinary extracellular vesicles and especially urinary exosomes are secreted by renal tubular epithelial cells and carry nucleic acids, proteins, and lipids; they can be easily detected in urine and provide diagnostic and pathophysiological information without an invasive tissue biopsy (11–13).

Exosomes have a size of 40–100 nm, are secreted by all cell types, and are composed of a lipid bilayer with membrane receptors and nucleic acids inside. They are involved in extracellular trafficking, differentiation, and survival, and could provide information about transcription in cells of urogenital tissue. HSD11B2 mRNA is expected to be present in urinary exosomes, considering the tubular localization of the enzyme; however, its presence in exosomes has not been documented so far. Some authors hypothesized the usefulness of detecting HSD11B2 or even other exosomal mRNAs for the study of the pathophysiological mechanisms of hypertensive diseases and in particular for the diagnosis of mineralocorticoid hypertension (14). Urinary exosomes are considered extremely valuable for diagnostic purposes as they are considered as kidney liquid biopsies (15) because urinary EVs (uEVs) are mainly derived from renal cells while circulating serum EVs, under physiological conditions, cannot cross the nephron barrier (12, 16). The investigation of the urinary exosomal expression of HSD11B2 can therefore open up novel perspectives in the complex diagnostic and prognostic processes in non-classic forms of EH and especially in AME. In this context, our study was aimed to identify an accurate method to detect and quantify HSD11B2 mRNA from urinary exosomes in samples from family members affected by AME (17) and in EH patients to investigate a possible relationship between urinary exosomal HSD11B2 mRNA, steroid ratio, HSD11B2 662 C>G genotype (corresponding to a 221 Ala>Gly substitution in the amino acid sequence), and hypertension status.

## Materials and Methods

In this observational case–control study, we compared HSD11B2 exosomal mRNA copy numbers and urinary steroid ratios measured by two different methods, in AME family members and hypertensive subjects.

### Subjects Selection

To study HSD11B2 urinary exosomal mRNA, two groups of subjects were enrolled: (1) hypertensive patients and (2) members of a family affected by AME as previously described (17).

Blood sample for biochemical routine parameters, 24-h urine, and second morning urine samples were collected for each subject. Second morning urine was obtained the same day of the collection of the 24-h urine vessel. A sterile container was given to patients who provided the second morning void directly at the outpatient clinic.

Measurement of biochemical, hormonal, and routine laboratory tests were performed at the laboratory of the Clinical Chemistry Institute of the Verona University Hospital. Plasma renin and aldosterone levels were measured by commercially available methods (Dia Sorin Diagnostics, Vercelli, Italy), as previously described (18).

The study was approved by the Ethics Committee of our Institution (Azienda Ospedaliera Universitaria Integrata, Verona, Italy) and patients gave their informed written consent after full explanation of the study.

#### Hypertensive Subjects

Patients were enrolled among those referring to the Hypertension Unit of the Verona University Hospital for resistant hypertension or for possible secondary causes of hypertension. After the exclusion of secondary forms of hypertension (such as nephro-parenchymal disease, primary aldosteronism, renovascular hypertension, cathecolamine excess, and cortisol excess) only patients for whom a diagnosis of EH was made were included in the study. Similarly, patients currently treated with glucocorticoids, or with a clinical history of previous glucocorticoid treatment, were excluded. None was taking licorice-containing sweets, or 11β-HSD2 inhibitors, such as cancer treatment dithiocarbamates (DTCs) or fungicides such as itraconazole, hydroxyitraconazole (OHI), and posaconazole (19, 20).

According to the study protocol, all patients had not taken any hypotensive drugs other than verapamil and/or alpha-blockers over the previous 4 weeks. Plasma samples for aldosterone and renin were obtained after at least 2 h in the upright position and a subsequent period of 10 min in the seated position. Blood samples for hormonal and routine parameters were collected after overnight fasting between 8:00 and 9:00 a.m. Biochemical parameters were determined and 24-h urine cortisol/cortisone metabolite assay was performed on 24-h collected urine.

Fourteen subjects with a definite diagnosis of EH (equal to the number of the AME family members included in the study) were selected based on the availability of biological samples for the evaluation of HSD211B exosomal mRNA.

#### AME Family Members

Fourteen members of a family previously studied for a story of AME syndrome (14) were enrolled for this study. The subjects were subdivided according to their HSD11B2 662 C>G genotype (homozygous, 662 GG; heterozygous, 662 AG; wild type, 662 AA) and phenotype [normotensive (N); hypertensive (H)]. Clinical and biochemical follow-up of the two probands was also available at approximately 3 years after the diagnosis.

The two homozygous probands were not taking antihypertensive drugs at the time of enrolment whereas the other family members with a previous diagnosis of hypertension were already on treatment. The two probands were followed over time, with clinical and biochemical evaluation.

### F/E and THF+Allo-THF/THE Ratio Measurement

Urinary free cortisol (F) and cortisone (E) quantification was performed using a validated liquid chromatography tandem mass spectrometry (LC-MS/MS) method, as extensively described elsewhere (21). Briefly, separation and quantification of both steroids was performed by using the MS urinary free cortisol/cortisone kit (ISBN-BSN, Castelleone, Italy) on a Nexera X2 series UHPLC (Shimadzu, Kyoto, Japan) coupled with a 4500 MD triple quadrupole MS (Sciex, Milan, Italy) detector. The mean intra- and inter-assay imprecision were between 1.7% and 11.3%

Total tetrahydro-cortisol (THF), 5α-tetrahydrocortisol (5α-THF), and tetrahydro-cortisone (THE) were quantified by gas chromatography–mass spectrometry (GC-MS) method, as previously described (17, 22). Instrumentation used comprised an Agilent Technologies 7890A gas chromatograph and an Agilent Technologies 5975C inert MSD detector (Agilent Technologies Inc., Santa Clara, CA, USA). The mean intra- and inter-assay imprecision were between 3.9% and 13% for both methods.

Either F/E or THF+allo-THF/THE was measured on 24-h collected urine.

### Urinary Extracellular Vesicles (Exosome) Isolation

Second morning urine samples from AME family members and patients affected by EH were collected, processed as previously described with minor modifications (23) and stored at −80°C. Second morning void was chosen as good option for UV isolation based on previous reports (24, 25). Samples were thawed at room temperature and extensively vortexed to increase exosome yield. Aliquots of 5 ml of urine were mixed with the same volume of a commercially available precipitating reagent (Total Exosome Isolation Reagent from urine; ThermoFisher Scientific, Waltham, Massachusetts, USA) and incubated for 1 h at RT according to the manufacturer’s instructions. Urine mixed with reagent were centrifuged at 10,000 *g* and 4°C for 1 h. The obtained pellet was used for analysis after careful removal of the supernatant. Vesicle size was checked by electron microscop.

### RNA Extraction

Total exosomal RNA was extracted by a commercial kit (PureLink RNA Micro kit; Invitrogen, Carlsbad, California, USA). According to the manufacturer’s instructions, RNA was extracted, purified, and eluted in a final volume of 15 µl and stored at −80°C.

Synthesis of cDNAs was carried out using a commercial kit (iScript Advanced cDNA synthesis kit for RT-PCR; Biorad, Hercules, California, USA) and a preamplification step was included (obtained by the use of SsoAdvanced PreAmp Supermix; Biorad, Hercules, California, USA) to improve mRNA detection and measurement. Preamplification primer mix included the genes HSD11B2 and β-2 microglobulin (B2M).

### Reverse Transcription-Droplet Digital Polymerase Chain Reaction (ddPCR)

Droplet Digital PCR was performed at the Genomics and Transcriptomics Platform of “Centro Piattaforme tecnologiche” of the University of Verona by the use of Bio-Rad QX200 Droplet digital PCR system (Bio-Rad). Reactions and processing of the samples were obtained following the manufacturer’s instructions. In brief, the mix completed with cDNA samples were loaded with a multichannel pipette into a droplet generator cartridge and 70 µl of oils was added to the lower wells and the cartridge or reaction plates containing the samples was placed into a QX200 Automatic Droplet Generator (Bio-Rad) to produce the individual droplets. Each reaction was partitioned into ~20,000 nanoliter-sized droplets. Procedures were standardized and performed with high care. After the PCR was completed, the sealed plate containing the droplets was loaded in a QX200 droplet reader for the detection of completed PCR reaction individual droplets. Data were analyzed with Quanta Soft Software version 1.7.4.0917 (Bio-Rad) with the thresholds for detection set manually based on results from negative control wells containing water instead of RNA. During all the preparation, and in particular for the steps of droplet generation and droplet handling, high accuracy was required to preserve droplet integrity. The detection of HSD11B2 RNA was assumed valid only if the housekeeping gene B2M was present. Optimization was evaluated by the separation between positives and negatives. qPCR results were calculated according to the standard curves. Mean values and standard deviations of ddPCR and qPCR were assessed using Student’s *t*-test. Reproducibility was tested with three independent samples and intra-assay coefficients of variation (CV) calculated for HSD11B2 and B2M mRNA were <5%. Statistical significance was achieved with *p*-value < 0.05. More details on RNA quantification are available in the Supplementary Material.

### Statistical Analysis

Data analysis for comparison of clinical/biochemical data was performed using the SPSS 24.0 for Windows (SPSS, Chicago, Illinois, USA). Quantitative data are expressed as median values with minimum–maximum range, while categorical variables were expressed as percentage. Continuous variables showing a skewed distribution were analyzed on log-transformed values. Correlations between continuous variables were assessed by Pearson’s test. Taking into account the very limited sample size of the different study subgroups (e.g., AME family members with different genotypes or EH patients analyzed according to exosomal HSD11B2 RNA copy number), all the comparisons are shown only as descriptive and not quantitative statistics.

## Results

**Figure 1:**
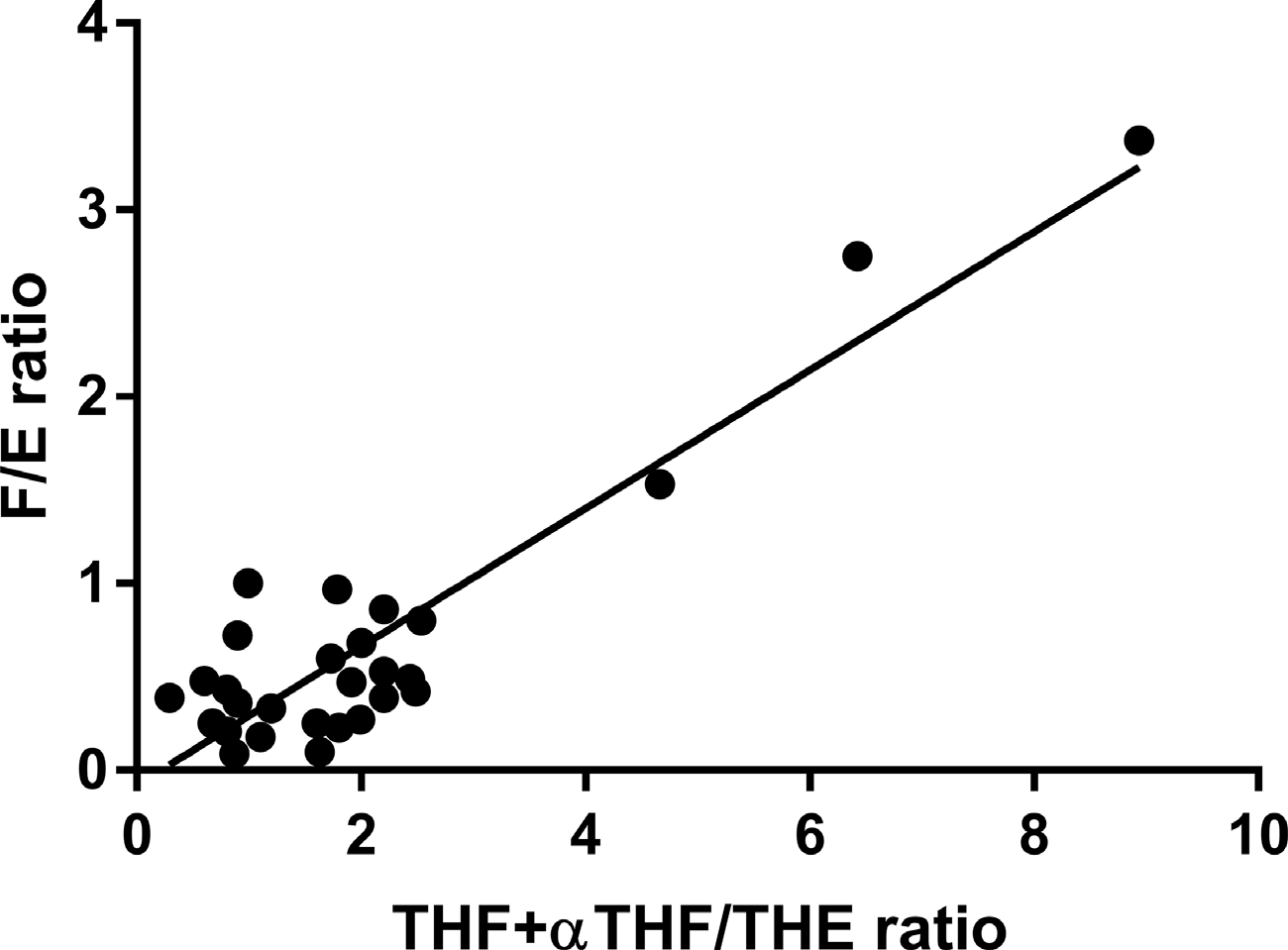
THF+allo-THF/THE and F/E ratio correlation in the complete study population (*n* = 28). *r* = 0.9134, *p* < 0.0001.

Subjects enrolled in the study were characterized in terms of hormonal and biochemical parameters. In particular, the members of the AME family presented the features shown in **Supplementary Table S1** detailed for each family member and in **Table 1** according to genotype. Family members affected by AME displayed the typical phenotype of the disease, with aldosterone, renin, and K lower in 221 GG subjects than in heterozygous 221 AG and wild-type 221 AA subjects as shown in Supplementary Figure S1. Characteristics of EH patients are reported in **Table 2**. THF+aTHF/THE and F/E ratios showed a good correlation in the whole population. As illustrated in **Figure 1**, there was a positive significant correlation between the two parameters (*r* = 0.9134, *p* < 0.0001).

**Table 1:** Characteristics of the AME family members according to genotype*.

	221 GG	221 AG	221 AA
**Age**	10 (7–13)	51 (25–81)	49 (20–79)
**Hypertension (%)**	100	57.1	20
**THF+aTHF/THE ratio**	7.66 (6.41–8.91)	1.91 (1.60–2.54)	0.99 (0.29–1.80)
**F/E ratio**	3.06 (2.75–3.37)	0.49 (0.25–0.97)	0.16 (0.09–0.39)
**K (mmol/L)**	2.94 (2.60–3.28)	3.97 (3.12–4.33)	3.9 (3.64–4.80)
**P-Renin (pg/ml)**	2.85 (2.46–3.24)	8.46 (0.84–15.96)	8.23 (6.06–40.40)
**P-Aldosterone (pg/ml)**	<15	106 (15–134)	128 (70–185)
**P-creatinine (mg/dl)**	0.56 (0.44–0.68)	0.76 (0.68–0.94)	0.75 (0.71–0.99)
**P-cortisol (µg/dl)**	16.25 (14.80–17.70)	11.1 (10.20–18.30)	12.7 (9–22)
**ACTH (pg/ml)**	53.4	19 (10.70–25.50)	17.3 (0.76–51)

*Data are expressed as median with minimum–maximum range.

**Table 2:** Biochemical and clinical features of EH patients*.

**Age**	49 (22–68)
**Gender (M/F)%**	36/64
**THF+aTHF/THE ratio**	1.15 (0.6–4.67)
**F/E ratio**	0.41 (0.18–1.63)
**K (mmol/L)**	3.86 (3.19–4.28)
**P-Renin (pg/ml)**	9.71 (1.2–33.16)
**P-aldosterone (pg/ml)**	185 (103–456)
**P-creatinine (mg/dl)**	0.82 (0.44–1.11)
**P-cortisol (µg/dl)**	13.95 (7–25.6)
**ACTH (pg/ml)**	15.96 (4.82–26.7)

*Data are expressed as median with minimum-maximum range.

**Figure 2:**
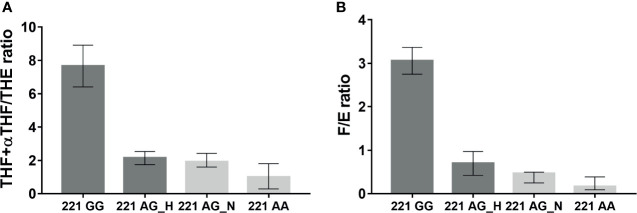
THF+allo-THF/THE **(A)** and F/E **(B)** ratios in family affected by AME according to genotype (GG homozygous, AG heterozygous, AA wild type) and hypertensive status (H, Hypertensive; N, Normotensive).

Values of THF+αTHF/THE ratio measured in the AME family were higher in 221 GG subjects (*n* = 2, 7.66, range 6.41–8.91) than those in the other groups, 221 AG_H (*n* = 4, 2.14, range 1.74–2.54), 221 AG_N (*n* = 3, 1.91, range 1.6–2.43), and 221 AA (*n* = 4, 0.99, range 0.29-1.8) (**Figure 2A**). In parallel, the highest F/E ratio values were found in 221 GG subjects (*n* = 2, 3.06, range 2.75–3.37) and the lowest values were found in 221 AA subjects (*n* = 5, 0.16, range 0.09-0.39) (**Figure 2B**). The F/E ratio values also showed a decreasing trend according to hypertensive status, 221 AG_H (*n* = 4, 0.70, range 0.42–0.97), 221 AG_N (*n* = 2, 0.47, range 0.25–0.49) (**Figure 2** and **Table 1**).

In EH patients, mean values for THF+allo-THF/THE and F/E were 1.59 ± 1.09 and 0.52 ± 0.35, respectively (**Table 2**).

**Figure 3:**
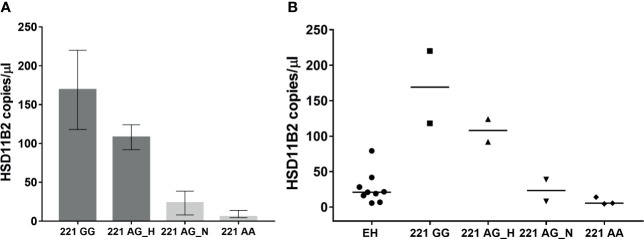
HSD11B2 exosomal mRNA (copies/µl) in AME subject according to genotype (GG homozygous, AG heterozygous, AA wild type) and hypertensive status (H, Hypertensive; N, Normotensive) **(A)** and in all subjects including EH **(B)**.

Urine samples were processed for exosome extraction and HSD11B2 exosomal mRNA was measured by ddPCR. Method optimization and standardization was performed in order to obtain reliable results. Samples positive for the presence of both the housekeeping gene and the target gene were used for further analysis. HSD11B2 mRNA was expressed as copies/µl. The distribution of the enzyme in the different experimental groups investigated is shown in **Figure 2**. In the AME family, HSD11B2 exosomal mRNA was higher in mutated subject 221 GG (169, range 118–220 copies/µl) and progressively lower in 221 AG_Hypertensive (108, range 92–124), 221 AG_Normotensive (23.35, range 8–38.7), and wild-type 221 AA subjects (5.5, range 4.5–14). EH patients had HSD11B2 exosomal mRNA (20.9, range 5.6–79.2) lower than homozygous and heterozygous AG H (**Figure 3**). HSD11B2 mRNA data were available only for nine AME and nine EH subjects; as for the other subjects, the housekeeping gene was undetectable.

**Figure 4:**
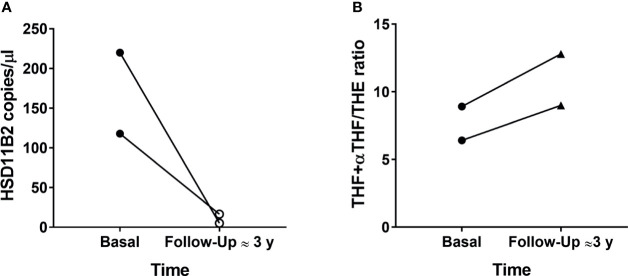
HSD11B2 exosomal mRNA (copies/µl) **(A)** and THF+allo-THF/THE ratio **(B)** in the two probands 221 GG mutated at enrolment and follow-up (≈3 years).

Furthermore, the two probands of the AME family, homozygous for the GG allele, were assessed also at 3 years follow up. The analysis revealed that HSD11B2 exosomal mRNA levels were greatly reduced, −86% with a decrease from 118 copies/µl to 16.6 copies/µl, and −98%, from 220 copies/µl to 5.1 copies/µl. By contrast, levels of THF+allo-THF/THE ratios were higher than those at the time of the enrolment, from 8.91 to 12.8 and from 6.41 to 9, respectively (**Figures 4A, B**). Other clinical–biochemical parameters improved from baseline, with a better blood pressure control (in relation to antihypertensive therapy including eplerenone, a MR receptor antagonist) and normalization of kalemia. We then analyzed the correlation between HSD11B2 mRNA copies and other biochemical parameters, in particular THF+αTHF/THE and F/E ratio.

**Figure 5:**
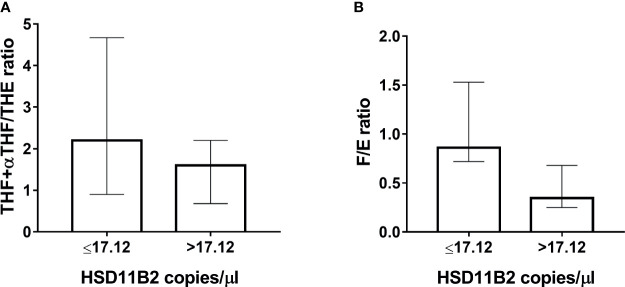
Classification of EH patients in low and high HSD11B2 mRNA when below or higher than the first tertile (17.12 copies/µl), respectively. **(A)** THF+allo-THF/THE ratio. **(B)** F/E ratio.

**Figure 6:**
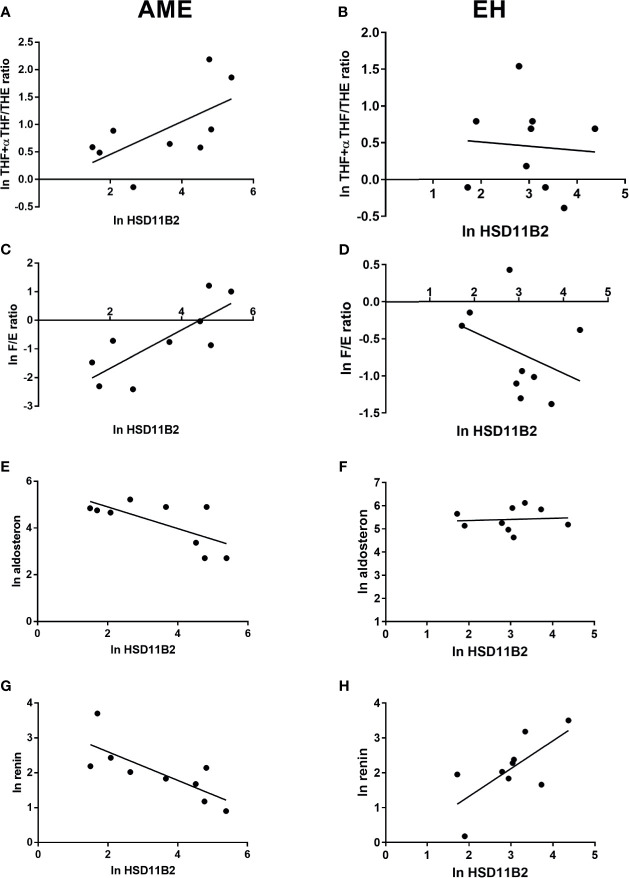
HSD11B2 exosomal mRNA levels and steroid ratio and aldosterone and renin correlations in AME subjects (on the left) and in EH patients (on the right). 11β-HSD2 exosomal mRNA showed a positive correlation with THF+allo-THF/THE ratio in AME subjects, *r* = 0.6266, *p*-value = 0.0710 **(A)** and an inverse correlation in EH patients, *r* = −0.07793, *p*-value = 0.8420 **(B)**. Similar trend was found between 11β-HSD2 exosomal mRNA and F/E ratio in AME, *r* = 0.7825, *p*-value = 0.0127 **(C)** and inverse in EH, *r* = −0.3762, *p*-value = 0.3184 **(D)**. On the contrary, an inverse and significant correlation was found between 11β-HSD2 exosomal mRNA and aldosterone and renin in AME and a positive significance for renin in EH, *r* = −0.6921, *p*-value = 0.0388 **(E)** and *r* = −0.7649, *p*-value = 0.00164 **(G)**, *r* = 0.692, *p*-value = 0.039 **(H)**, respectively. No correlation was found in EH for 11β-HSD2 exosomal mRNA and aldosterone, *r* = 0.081, *p*-value = 0.8359 **(F)**.

The EH population was divided into two subgroups according to HSD11B2 mRNA copy number: the first group within the first tertile and the second group above the first tertile value (17.12 copies/µl) (**Figure 5A**). Both ratios were higher in the EH group with a lower HSD11B2 mRNA copy number (2.20, range 0.9–4.67 *vs.* 1.6, range 0.68–2.2 for THF+αTHF/THE and 0.86, range 0.72–1.53 *vs.* 0.34, range 0.25–0.68 for F/E, respectively) (**Figure 5B**). The two EH subgroups differed also in K concentrations and age, as reported in **Table 3**. The main correlations studied between HSD11B2 mRNA and biochemical parameters are illustrated in **Figure 6**, where AME subjects are represented on the left and EH patients are represented on the right side of the figure. In the AME family, both urinary steroid ratios correlated positively with HSD11B2 mRNA (**Figures 6A, C**); by contrast, the correlation was negative in the EH population (**Figures 6B, D**), even if statistical significance was reached only for F/E in the AME subjects (**Figure 6C**, *r* = 0.7825, *p*-value = 0.0127). In AME, the mRNA exosomal copies of HSD11B2 significantly inversely correlated with aldosterone and renin (*r* = −0.6646, *p*-value = 0.0184 and *r* = −0.7649, *p*-value = 0.0164), respectively (**Figures 6E–G**). In EH patients, aldosterone levels did not show any correlation with HSD11B2 mRNA levels (**Figure 6F**), while renin values displayed a positive correlation with HSD11B2 mRNA levels (*r* = 0.692, *p* = 0.039 **Figure 6H**). In addition, when data were analyzed for a possible influence of age and BMI on both urinary steroid ratios in the whole population, no significant correlation was observed by Pearson’s test. Furthermore, steroid ratios were also evaluated in relation to aldosterone and renin values either in AME or in EH subjects (**Supplementary Figure S2**). THF+αTHF/THE ratio showed an inverse correlation with aldosterone (*r* = −0.555, *p*-value = 0.0394) and an inverse but non-significant correlation with renin (*r* = −0.4491, *p*-value = 0.1237) in the AME family (**Supplementary Figure 2A**) despite the fact that, in EH, no correlation was found (**Supplementary Figures 2A, B**). Similar trends were observed for F/E ratio with renin and aldosterone levels; a significant negative correlation was in fact found in AME (*r* = −0.6225, *p*-value = 0.0231; *r* = −0.666, *p*-value = 0.0093, respectively) while there was no significant correlation in EH patients (**Supplementary Figures 2C, D**).

**Table 3:** Characteristics of EH patients divided into low and high HSD11B2 mRNA copy number*.

	<17.12	≥17.12
Age	59 (41–78)	44 (33–53)
Gender (M/F) %	33/67	50/50
THF+aTHF/THE ratio	2.2 (0.90–4.67)	1.60 (0.68–2.20)
F/E ratio	0.86 (0.72–1.53)	0.34 (0.25–0.68)
K (mmol/L)	3.40 (3.19–3.68)	4.01 (3.35–4.26)
P-Renin (pg/ml)	7.04 (1.20–7.60)	10.3 (5.28–33.16)
P-Aldosterone (pg/ml)	190 (171–283)	261 (103–456)
P-creatinine (mg/dl)	0.86 (0.59–1.11)	0.81 (0.44–0.99)
P-cortisol (µg/dl)	13.25 (11.7–15.60)	14 (7–25.6)
ACTH (pg/ml)	19.86 (14.7–26.7)	13.65 (4.82–24)

*Data are expressed as median with minimum-maximum range.

## Discussion

To the best of our knowledge, this is the first report showing that HSD11B2 mRNA is detectable in urinary exosomes and correlates with the 11β-HSD2 activity estimated by urinary steroid ratios. The results highlight, moreover, that the urinary exosomal HSD11B2 mRNA expression varies according to the HSD11B2 662C>G genotype in an AME family, suggesting a potential use of it as a molecular marker of hypertensive disease, due to an impaired 11β-HSD2 enzyme function.

The-11β-HSD2 is a crucial enzyme for the modulation of MR activation, a key receptor at the kidney cellular level for the regulation of sodium and water balance in arterial hypertension. Cortisol inactivation by 11β-HSD2 mediates the ligand selectivity of aldosterone for MR, and an impaired enzymatic activity led to the cortisol activation of MR in AME disease.

The study of 11β-HSD2 enzyme is of interest because, even if AME is a rare disease, with about 100 cases and overall 50 mutations described in the literature, a partial deficiency of this enzyme was proposed also in other conditions, i.e., arterial hypertension and renal failure. A moderate impairment of 11β-HSD2 was reported in some EH patients, and a number of polymorphisms or genetic mutations with a mild influence on 11β-HSD2 activity were described in EH (7, 26, 27). Moreover, in relatives of AME patients, a mild form of hypertension, indistinguishable from EH, was often described (28, 29). We previously demonstrated that a number of EH patients and glucocorticoid-treated patients shared a similar phenotype, characterized by both arterial hypertension and elevated urinary THF+aTHF/THE ratio (22). Recent research suggests that milder forms of AME could be present in the general population, and hypertensive patients were mislabeled as idiopathic. This condition was previously described in literature as AME type II (1, 7, 30), and more recently described as “non-classic” AME, with a much higher prevalence (7.1%) than classic AME (8, 9, 31).

In humans, the 11β-HSD iso-enzyme 11β-HSD1 (converting the inactive cortisone into the active cortisol) is widely distributed but it is mostly abundant in liver and adipose tissue, whereas the 11β-HSD2 iso-enzyme (inactivating cortisol to cortisone) is primarily expressed in MR target tissues, i.e., kidney, colon, and salivary gland. To study the functional role of 11β-HSD2 in the regulation of water balance, the most critical issue is that the kidney cannot be easily available unless a renal biopsy, i.e., an invasive procedure is made. The 11β-HSD2 enzyme activity is, therefore, usually estimated by a surrogate marker, i.e., the urinary cortisol-to-cortisone metabolite ratios. As a proof of this assumption in a cohort of patients undergoing renal biopsy because of suspected underlying renal disease, it was demonstrated that renal 11β-HSD2 mRNA expression relates to both urinary cortisol metabolite ratios (THF+aTHF/THE and F/E). Moreover, a decreased HSD11B2 expression was observed among the patients with worse renal function (32). In different patients, such as AME and EH, similar results have never been obtained so that the same relationships between cortisol metabolites ratios and 11β-HSD2 activity is far from being firmly proved. In this context, we hypothesized that urinary extracellular vesicles and exosomes might represent a useful tool for investigation and a possible source of information. Since the 11β-HSD2 enzyme is expressed in the kidney, we hypothesized that HSD11B2 mRNA is part of the urinary exosome cargo and therefore a possible liquid biomarker of disease related to 11β-HSD2 enzyme dysregulation. The results here presented confirm our hypothesis.

We demonstrated that HSD11B2 mRNA is detectable in urinary exosomes both in patients with AME syndrome and in patients with EH. The relationship with clinical–biochemical parameters are different in the two conditions. In the AME family, the expression of HSD11B2 mRNA varied according to the 662 C>G genotype: the homozygous probands showed the highest levels, the heterozygous subjects showed intermediate levels, and the wild-type subjects showed the lowest levels. Among heterozygous carriers, hypertensive subjects had higher HSD11B2 exosomal mRNA than normotensives. These data suggest the following: (i) higher expression of the gene when the enzymatic activity is mostly reduced, at least as estimated by urinary steroid ratios (urinary THF/THE and THF+5αTHF/THE ratios); (ii) 11β-HSD2 mRNA is inversely related to the renin and aldosterone concentrations, mirroring a pronounced inhibition of the renin–aldosterone axis. The first point may be explained by an increased rate of transcription of the mutated gene as a feedback mechanism to compensate for the reduced enzymatic activity. However, this effect could be specific for the 662 C>G variant, while other mutations at different points of the gene sequence could have inhibitory consequences on the transcription process with reduced or absent mRNA.

The mechanisms previously investigated of the modulation of 11β-HSD2 gene expression were at pre- and post-transcriptional levels: the presence of polymorphisms, variations in microsatellite regions (33, 34), and epigenetic modifications, i.e., differentially expressed miRNAs (35, 36) and CpG methylation at the HSD11B2 promoter region (17, 37–39).

DNA methylation is the main epigenetic feature in mammalian cells, leading to a gene transcriptional regulation by transcriptional repression when the gene promoter site is hypermethylated (40). In a cohort of patients treated with glucocorticoid with high urinary THF+aTHF/THE, HSD11B2 promoter methylation in peripheral leukocytes was associated with the development of hypertension, suggesting a possible role of a modulation of HSD11B2 gene expression in the pathogenesis of steroid-induced hypertension (39). In the same AME family investigated in this study, we previously demonstrated a lower HSD11B2 promoter methylation in the two affected probands, compared to the wild-type individuals (17), and this result is consistent with the present finding of a great amount of HSD11B2 exosomal mRNA measured in the two probands.

A further indirect demonstration of the role of a gene expression modulation of HSD11B2 related to the entity of MR activation derives from the data of the two probands at follow-up. At baseline, when the two patients were free from specific therapy, with a florid disease characterized by low-renin, low-aldosterone hypokalemic hypertension, we observed a high exosomal HSD11B2 mRNA expression. After a long period of antihypertensive therapy including MR antagonist (eplerenone), along with a good blood pressure control and normalization of renin and kalemia, we observed a clear reduction in exosomal HSD11B2 mRNA. If HSD11B2 gene expression at baseline was increased in order to compensate for the excessive MR activation, a therapy-mediated MR inhibition also reduced HSD11B2 mRNA. Interestingly, the higher THF+aTHF/THE ratio as compared to baseline observed after eplerenone treatment in the two boys is consistent with the finding of a lower exosomal HSD11B2 gene expression.

The enzymatic activity of 11β-HSD2 is estimated by either urinary THF+aTHF/THE or serum or urinary F/E ratio. It was hypothesized that F/E may be a better marker of the activity of 11β-HSD2 isoenzyme, while THF+5αTHF/THE may represent a proxy of global HSD11B2 activity (i.e., both 11β-HSD1 and 11β-HSD2 isoforms) (7, 41–46). Which parameter is better to assess 11β−HSD2 activity in humans remains unclear. In the population of this study, F/E has a better correlation with exosomal HSD11B2 gene expression than the THF+aTHF/THE ratio: in the AME family, the positive correlation between HSD11B2 exosomal mRNA was positive for both urinary ratios, but reached the statistical significance only for the F/E, and the negative correlation between HSD11B2 exosomal mRNA and renin was significant only for the F/E ratio. In the EH population, U/F better discriminated patients according to median values of HSD11B2 mRNA than the THF+aTHF/THE ratio. However, because of the limited sample size, we cannot definitively state that urinary F/E is better than the THF+aTHF/THE ratio in estimating enzymatic 11β-HSD2 activity. Further studies are needed to confirm our data.

A further strength of the study is the accuracy of the method to quantify the urinary HSD11B2 mRNA expression since we utilized an advanced and sensitive novel technology, namely, the ddPCR (47) that is particularly suited for extremely low-target quantitation from variably contaminated samples as it is based on the partitioning of the PCR reaction into thousands of individual reaction vessels prior to amplification and on the acquisition of data at reaction end point. DdPCR is thus able to give more precise and reproducible data than qPCR (48).

The present study has some limitations that need to be acknowledged. The study sample is very limited and, therefore, mostly descriptive, which implies that they should be interpreted with caution. We also recognize the substantial overlap in exosomal HSD11B2 RNA copy number between different subgroups, including 221 GG homozygotes and hypertensive 221 AG heterozygotes, which limits the diagnostic value of such assay. Moreover, the comparison between AME family members and EH subjects is limited by the lack of age and sex matching. Digital PCR has proven to be the right tool for investigating HSD11B2 mRNA, as other approaches were not successful in allowing the detection of such a minimal amount of material such as the mRNA present in UVs, a limitation to get enough mRNA to analyze all samples, even after the necessary step of preamplification. Further studies are indeed required to confirm these findings in a larger data set.

Despite the study limitations, our results appear biologically plausible and could be seen as a potential proof of concept about the role of 11βHSD2 activity in mineralocorticoid hypertension.

HSD11B2 mRNA is detectable and quantifiable in urinary exosomes by ddPCR technology. In AME family members (662 C>G genotype), we observed an increased HSD11B2 expression in homozygous as compared to heterozygous and wild types and a positive correlation between exosomal mRNA and the 11βHSD2 enzyme activity as estimated by the urinary steroid ratios, i.e., opposite to the trend observed in EH patients. The overexpression of HSD11B2 mRNA is a possible mechanism of compensation of the enzyme deficiency. The study of exosomal expression is a useful tool to investigate 11β-HSD2 functional activity in kidney through the analysis of exosomal mRNA cargo in mineralocorticoid hypertension.

The present study, moreover, adds to the current knowledge and understanding that it is possible to perform a kidney tissue gene expression analysis simply by evaluating a urine sample. It is also possible to speculate that also other genes of interest related to diseases with a renal involvement could be investigated and provide novel insights for novel perspectives in clinical approaches.

## Data Availability Statement

The datasets generated for this study will not be made publicly available due to privacy policy. Requests to access the datasets should be directed to FP, francesca.pizzolo@univr.it.

## Ethics Statement

The studies involving human participants were reviewed and approved by Ethics Committee of our Institution (Azienda Ospedaliera Universitaria Integrata, Verona, Italy). The patients/participants provided their written informed consent to participate in this study.

## Author Contributions

Conceived and designed research: SF, FP, and OO. Performed experiments: LB, FM DD, ED, SU, and MV. Analyzed data: LB, DD, AC, FM, and SU. Interpreted results of experiments: OO, FP, SF, NM, and ED. Prepared figures: DD and NM. Drafted the manuscript: FP, AC, and DD. All authors contributed to the article and approved the submitted version.

## Funding

The funders had no role in study design, data collection and analysis, decision to publish, or preparation of the manuscript. This research was funded by the University of Verona independent funding project “Ricerca di Base” to FP.

## Conflict of Interest

The authors declare that the research was conducted in the absence of any commercial or financial relationships that could be construed as a potential conflict of interest.

## Publisher’s Note

All claims expressed in this article are solely those of the authors and do not necessarily represent those of their affiliated organizations, or those of the publisher, the editors and the reviewers. Any product that may be evaluated in this article, or claim that may be made by its manufacturer, is not guaranteed or endorsed by the publisher.
