# Supplementary material for: Detection of Urinary Exosomal HSD11B2 mRNA Expression: A Useful Novel Tool for the Diagnostic Approach of Dysfunctional 11β-HSD2-Related Hypertension
Source: Front Endocrinol (Lausanne). 2021 Aug 23;12:681974. doi: 10.3389/fendo.2021.681974 (PMC8419411; doi:10.3389/fendo.2021.681974)
Supplement: Supplementary file 1 [file Presentation_1.pptx]

## Slide 1
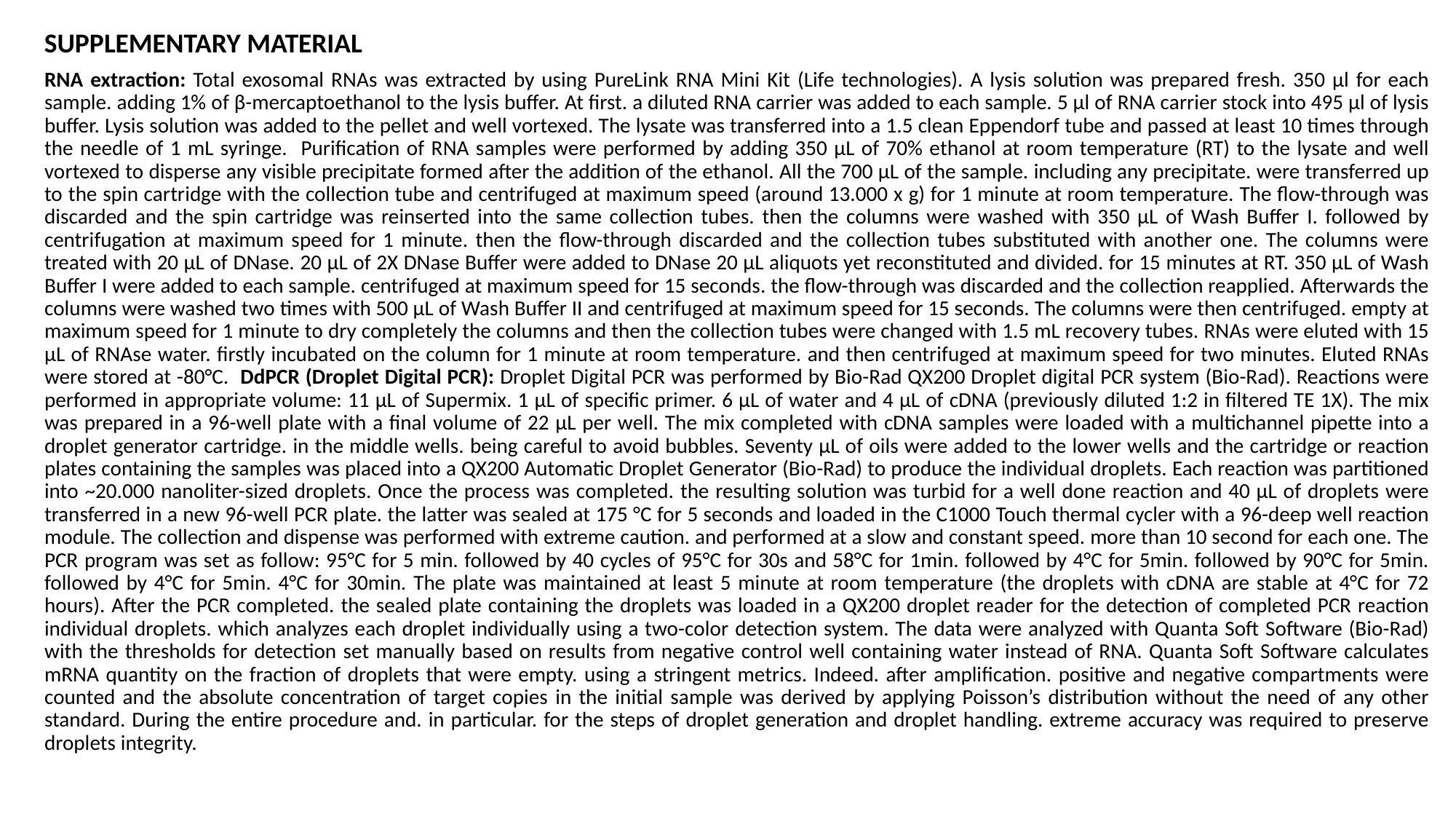

SUPPLEMENTARY MATERIAL
RNA extraction: Total exosomal RNAs was extracted by using PureLink RNA Mini Kit (Life technologies). A lysis solution was prepared fresh. 350 µl for each sample. adding 1% of β-mercaptoethanol to the lysis buffer. At first. a diluted RNA carrier was added to each sample. 5 µl of RNA carrier stock into 495 µl of lysis buffer. Lysis solution was added to the pellet and well vortexed. The lysate was transferred into a 1.5 clean Eppendorf tube and passed at least 10 times through the needle of 1 mL syringe. Purification of RNA samples were performed by adding 350 µL of 70% ethanol at room temperature (RT) to the lysate and well vortexed to disperse any visible precipitate formed after the addition of the ethanol. All the 700 µL of the sample. including any precipitate. were transferred up to the spin cartridge with the collection tube and centrifuged at maximum speed (around 13.000 x g) for 1 minute at room temperature. The flow-through was discarded and the spin cartridge was reinserted into the same collection tubes. then the columns were washed with 350 µL of Wash Buffer I. followed by centrifugation at maximum speed for 1 minute. then the flow-through discarded and the collection tubes substituted with another one. The columns were treated with 20 µL of DNase. 20 µL of 2X DNase Buffer were added to DNase 20 µL aliquots yet reconstituted and divided. for 15 minutes at RT. 350 µL of Wash Buffer I were added to each sample. centrifuged at maximum speed for 15 seconds. the flow-through was discarded and the collection reapplied. Afterwards the columns were washed two times with 500 µL of Wash Buffer II and centrifuged at maximum speed for 15 seconds. The columns were then centrifuged. empty at maximum speed for 1 minute to dry completely the columns and then the collection tubes were changed with 1.5 mL recovery tubes. RNAs were eluted with 15 µL of RNAse water. firstly incubated on the column for 1 minute at room temperature. and then centrifuged at maximum speed for two minutes. Eluted RNAs were stored at -80°C. DdPCR (Droplet Digital PCR): Droplet Digital PCR was performed by Bio-Rad QX200 Droplet digital PCR system (Bio-Rad). Reactions were performed in appropriate volume: 11 µL of Supermix. 1 µL of specific primer. 6 µL of water and 4 µL of cDNA (previously diluted 1:2 in filtered TE 1X). The mix was prepared in a 96-well plate with a final volume of 22 µL per well. The mix completed with cDNA samples were loaded with a multichannel pipette into a droplet generator cartridge. in the middle wells. being careful to avoid bubbles. Seventy µL of oils were added to the lower wells and the cartridge or reaction plates containing the samples was placed into a QX200 Automatic Droplet Generator (Bio-Rad) to produce the individual droplets. Each reaction was partitioned into ~20.000 nanoliter-sized droplets. Once the process was completed. the resulting solution was turbid for a well done reaction and 40 µL of droplets were transferred in a new 96-well PCR plate. the latter was sealed at 175 °C for 5 seconds and loaded in the C1000 Touch thermal cycler with a 96-deep well reaction module. The collection and dispense was performed with extreme caution. and performed at a slow and constant speed. more than 10 second for each one. The PCR program was set as follow: 95°C for 5 min. followed by 40 cycles of 95°C for 30s and 58°C for 1min. followed by 4°C for 5min. followed by 90°C for 5min. followed by 4°C for 5min. 4°C for 30min. The plate was maintained at least 5 minute at room temperature (the droplets with cDNA are stable at 4°C for 72 hours). After the PCR completed. the sealed plate containing the droplets was loaded in a QX200 droplet reader for the detection of completed PCR reaction individual droplets. which analyzes each droplet individually using a two-color detection system. The data were analyzed with Quanta Soft Software (Bio-Rad) with the thresholds for detection set manually based on results from negative control well containing water instead of RNA. Quanta Soft Software calculates mRNA quantity on the fraction of droplets that were empty. using a stringent metrics. Indeed. after amplification. positive and negative compartments were counted and the absolute concentration of target copies in the initial sample was derived by applying Poisson’s distribution without the need of any other standard. During the entire procedure and. in particular. for the steps of droplet generation and droplet handling. extreme accuracy was required to preserve droplets integrity.

## Slide 2
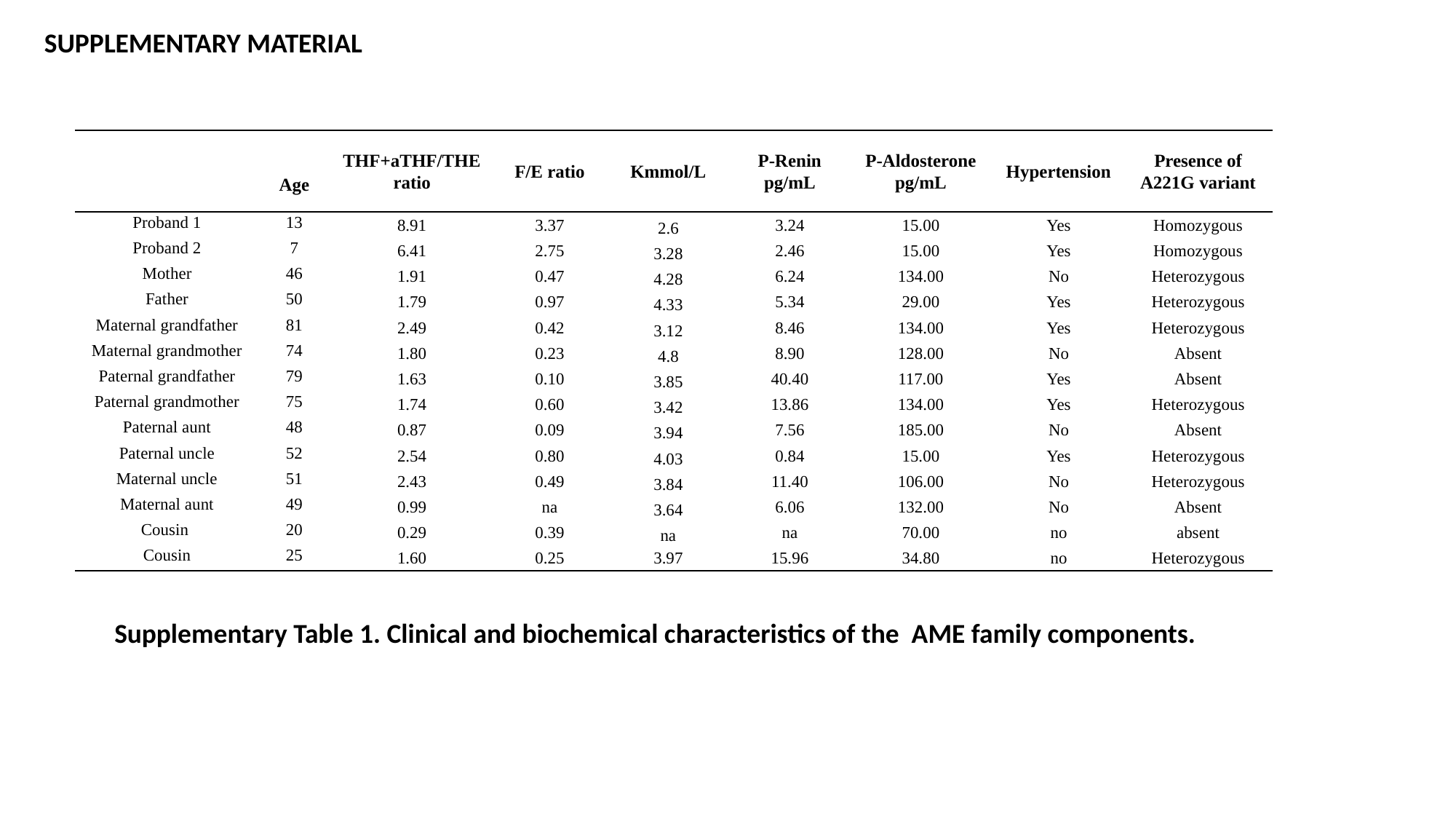

SUPPLEMENTARY MATERIAL
| | Age | THF+aTHF/THE ratio | F/E ratio | Kmmol/L | P-Renin pg/mL | P-Aldosterone pg/mL | Hypertension | Presence of A221G variant |
| --- | --- | --- | --- | --- | --- | --- | --- | --- |
| Proband 1 | 13 | 8.91 | 3.37 | 2.6 | 3.24 | 15.00 | Yes | Homozygous |
| Proband 2 | 7 | 6.41 | 2.75 | 3.28 | 2.46 | 15.00 | Yes | Homozygous |
| Mother | 46 | 1.91 | 0.47 | 4.28 | 6.24 | 134.00 | No | Heterozygous |
| Father | 50 | 1.79 | 0.97 | 4.33 | 5.34 | 29.00 | Yes | Heterozygous |
| Maternal grandfather | 81 | 2.49 | 0.42 | 3.12 | 8.46 | 134.00 | Yes | Heterozygous |
| Maternal grandmother | 74 | 1.80 | 0.23 | 4.8 | 8.90 | 128.00 | No | Absent |
| Paternal grandfather | 79 | 1.63 | 0.10 | 3.85 | 40.40 | 117.00 | Yes | Absent |
| Paternal grandmother | 75 | 1.74 | 0.60 | 3.42 | 13.86 | 134.00 | Yes | Heterozygous |
| Paternal aunt | 48 | 0.87 | 0.09 | 3.94 | 7.56 | 185.00 | No | Absent |
| Paternal uncle | 52 | 2.54 | 0.80 | 4.03 | 0.84 | 15.00 | Yes | Heterozygous |
| Maternal uncle | 51 | 2.43 | 0.49 | 3.84 | 11.40 | 106.00 | No | Heterozygous |
| Maternal aunt | 49 | 0.99 | na | 3.64 | 6.06 | 132.00 | No | Absent |
| Cousin | 20 | 0.29 | 0.39 | na | na | 70.00 | no | absent |
| Cousin | 25 | 1.60 | 0.25 | 3.97 | 15.96 | 34.80 | no | Heterozygous |
Supplementary Table 1. Clinical and biochemical characteristics of the AME family components.

## Slide 3
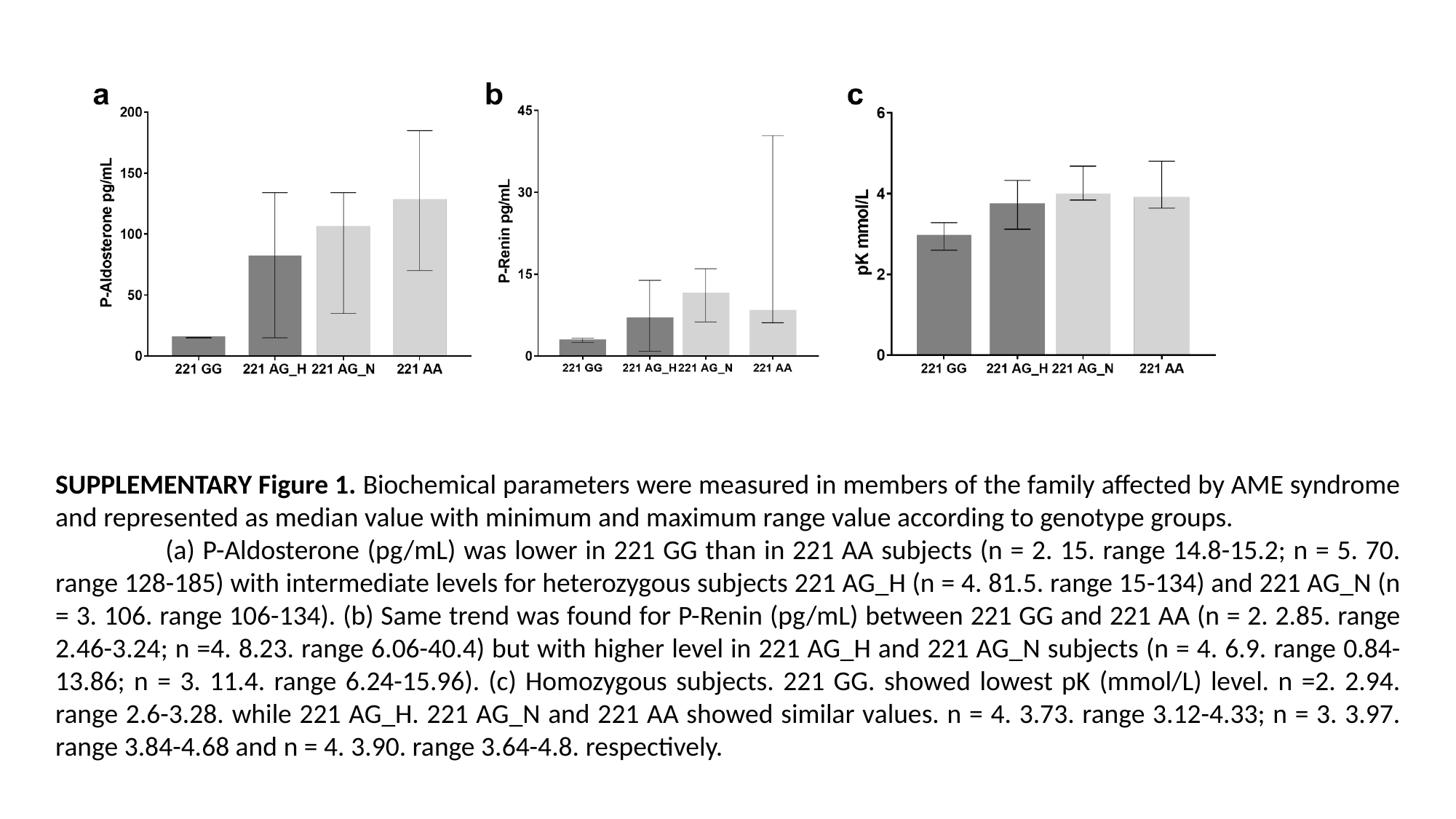

SUPPLEMENTARY Figure 1. Biochemical parameters were measured in members of the family affected by AME syndrome and represented as median value with minimum and maximum range value according to genotype groups. (a) P-Aldosterone (pg/mL) was lower in 221 GG than in 221 AA subjects (n = 2. 15. range 14.8-15.2; n = 5. 70. range 128-185) with intermediate levels for heterozygous subjects 221 AG_H (n = 4. 81.5. range 15-134) and 221 AG_N (n = 3. 106. range 106-134). (b) Same trend was found for P-Renin (pg/mL) between 221 GG and 221 AA (n = 2. 2.85. range 2.46-3.24; n =4. 8.23. range 6.06-40.4) but with higher level in 221 AG_H and 221 AG_N subjects (n = 4. 6.9. range 0.84-13.86; n = 3. 11.4. range 6.24-15.96). (c) Homozygous subjects. 221 GG. showed lowest pK (mmol/L) level. n =2. 2.94. range 2.6-3.28. while 221 AG_H. 221 AG_N and 221 AA showed similar values. n = 4. 3.73. range 3.12-4.33; n = 3. 3.97. range 3.84-4.68 and n = 4. 3.90. range 3.64-4.8. respectively.

## Slide 4
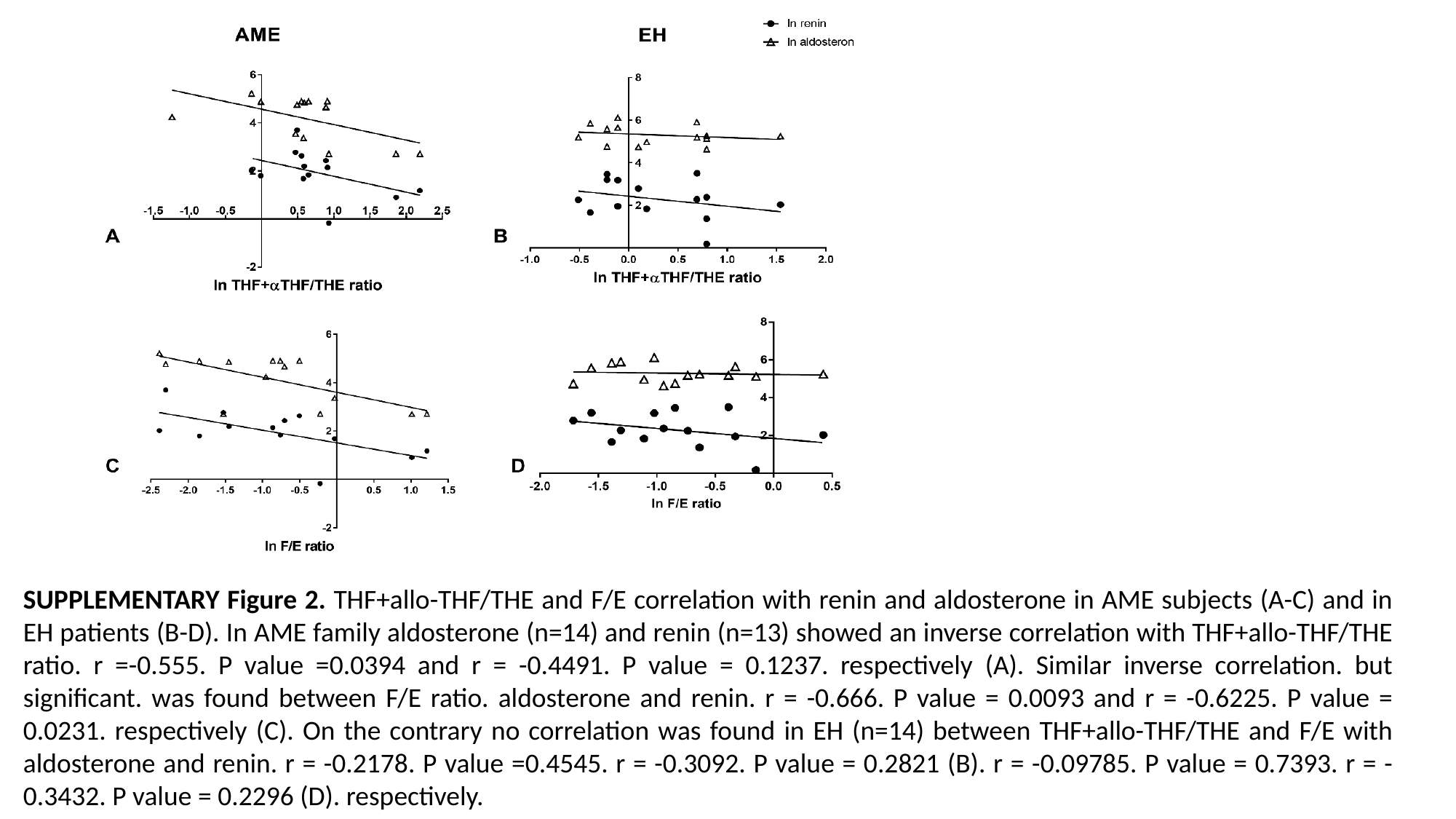

SUPPLEMENTARY Figure 2. THF+allo-THF/THE and F/E correlation with renin and aldosterone in AME subjects (A-C) and in EH patients (B-D). In AME family aldosterone (n=14) and renin (n=13) showed an inverse correlation with THF+allo-THF/THE ratio. r =-0.555. P value =0.0394 and r = -0.4491. P value = 0.1237. respectively (A). Similar inverse correlation. but significant. was found between F/E ratio. aldosterone and renin. r = -0.666. P value = 0.0093 and r = -0.6225. P value = 0.0231. respectively (C). On the contrary no correlation was found in EH (n=14) between THF+allo-THF/THE and F/E with aldosterone and renin. r = -0.2178. P value =0.4545. r = -0.3092. P value = 0.2821 (B). r = -0.09785. P value = 0.7393. r = -0.3432. P value = 0.2296 (D). respectively.
